# Investigating the Impact of Semi-Supervised Learning Methods to Improve the Quality of Diagnosis of Retinal Diseases from OCT Images

**DOI:** 10.3390/diagnostics16050656

**Published:** 2026-02-25

**Authors:** Armin Alizadeh, Ahmad Alenezi, Nastaran Khakestari, Yashar Amizadeh, Ata Jodeiri

**Affiliations:** 1 Department of Biomedical Engineering, Faculty of Advanced Medical Sciences, Tabriz University of Medical Sciences, Tabriz 51656, Iran; 2 Radiologic Sciences Department, Kuwait University, Kuwait City 31470, Kuwait; 3 Eye Surgery Department, Mehr Hospital, Tabriz 51656, Iran

**Keywords:** semi supervised learning, age-related macular degeneration, optical coherence tomography

## Abstract

**Background**: Age-related Macular Degeneration (AMD) is a leading cause of irreversible vision loss, particularly in the elderly. Optical Coherence Tomography (OCT), a noninvasive imaging modality, is widely used for retinal disease detection. However, the limited availability of labeled OCT datasets poses a significant challenge, making semi-supervised learning a promising approach. This study introduces a novel Iterative Teacher-Student (ITS) framework, which refines pseudo-labeling strategies to improve AMD detection accuracy, particularly in low-data scenarios. **Methods**: Initially, an optimal supervised model based on EfficientNet was developed to classify AMD using a dataset from Noor Eye Hospital, consisting of 16,822 OCT images. The dataset size was then progressively reduced to 70%, 50%, 20%, and 5% to evaluate model performance under data scarcity. Unlike conventional semi-supervised learning approaches, our ITS framework iteratively refines pseudo-labels, ensuring more reliable knowledge transfer from teacher to student models. **Results**: The optimized supervised model achieved 87.14% accuracy in AMD classification. As dataset size decreased to 20% and 5%, accuracy declined to 77.05% and 54.78%, respectively. Implementing the ITS framework improved accuracy to 88.56% at 20% and 64.15% at 5%, outperforming traditional semi-supervised methods. **Conclusions**: This study highlights the potential of semi-supervised learning, particularly our iterative teacher-student approach, to enhance AMD detection when labeled OCT data are scarce. The proposed framework introduces a novel iterative refinement strategy, which can serve as a foundation for future research in retinal disease diagnosis with limited labeled datasets.

## 1. Introduction

Retinal diseases encompass a range of conditions that can significantly impair vision and even lead to blindness. Retinopathies, such as glaucoma, macular holes, diabetic macular edema (DME), and age-related macular degeneration (AMD), often result in complications like macular edema and retinal detachment. AMD, a complex retinal disorder, is one of the leading causes of irreversible vision loss in industrialized countries, particularly affecting individuals over the age of 60 [1,2]. AMD is clinically categorized into two main subtypes: dry (non-exudative) and wet (exudative). In the early stages of dry AMD, yellowish-white deposits known as drusen form between the retinal pigment epithelium (RPE) and Bruch’s membrane (BM), marking the onset of the disease [3,4]. As these deposits accumulate, choroidal capillaries extend into the RPE and BM, leading to pathological changes characterized by abnormal choroidal neovascularization (CNV) beneath the retina. This progression can cause exudation, bleeding, scarring, and eventual loss of central vision [5,6,7]. The introduction of anti-VEGF therapies has significantly improved outcomes for wet AMD, helping to preserve visual acuity [1,8,9]. Early detection, however, remains crucial for preventing advanced retinal damage and reducing treatment costs.

**Figure 1:**
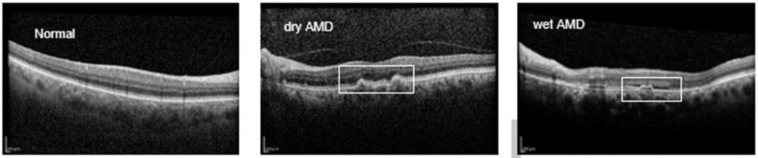
Sample OCT images of normal, dry AMD, and wet AMD eyes from the Noor Eye Hospital dataset. The white box highlights key features distinguishing these conditions.

Retinal disorders are commonly diagnosed using imaging techniques such as fundus photography, fluorescein angiography, and optical coherence tomography (OCT and OCTA) [5]. OCT, a noninvasive and noncontact modality, captures high-resolution, cross-sectional images of retinal layers through low-coherence light. Its high speed, resolution, and nondestructive imaging capabilities make OCT a valuable tool for diagnosing and monitoring retinal diseases [10,11]. OCT’s utility extends beyond ophthalmology; recent studies have highlighted its relevance in detecting neurological conditions like Alzheimer’s, multiple sclerosis, and Parkinson’s disease [12,13,14]. However, OCT interpretation poses challenges. As the population ages and AMD and other chronic retinal conditions increase, the demand for OCT evaluations is straining healthcare systems. Manual OCT analysis, especially for detecting subtle or early-stage lesions, presents significant challenges, as it can be both complex and prone to misinterpretation, particularly for conditions lacking distinct visual markers in the retina. Additionally, the process can be time-consuming, with notable delays in obtaining tests, reporting results, and associated costs, which may place a burden on patients and healthcare systems. Limited data availability for rare conditions further exacerbates the difficulty in achieving accurate diagnosis and effective treatment planning. These challenges highlight the limitations of relying solely on traditional manual OCT analysis to address the growing demands of retinal care. Figure 1 provides an illustration of OCT B-scans for AMD cases.

Artificial Intelligence (AI) encompasses technologies that simulate human cognitive functions, enabling machines to perform tasks requiring human-like understanding and pattern recognition Within AI, Machine Learning (ML) focuses on developing algorithms that learn from data, allowing systems to make predictions or decisions without explicit programming [15,16,17].

AI has made significant strides in multi-disease classification for healthcare, particularly in medical image analysis [18,19]. In ophthalmology, DL models have shown remarkable accuracy in detecting AMD-related lesions using OCT images, with notable studies advancing this application [6,10,20]. For example, He et al. proposed a two-stage DL model combining ResNet-50 with a local outlier factor algorithm to detect AMD from OCT volume scans, achieving high accuracy and AUC [21]. Alenezi et al. utilized a weighted fusion approach to combine outputs from models like ResNet and EfficientNet with attention mechanisms, achieving 91.88% accuracy in AMD classification [22]. SL models have also demonstrated efficacy across various tasks in OCT image analysis, including automated classification and segmentation of lesions [23,24]. Wang et al. used DenseNet, ResNet, and other architectures to classify OCT images into AMD, DME, and normal classes [25]. Meanwhile, Zapata et al. deployed a 24-layer CNN to differentiate between AMD and glaucomatous optic neuropathy using fundus and OCT images [26]. Other models, such as Dong et al.’s joint CNN detector using Yolov3, identified AMD among 11 retinal conditions with 88% sensitivity and 98% specificity [27]. In another study, Mathews et al. used a lightweight CNN to classify OCT images with 100% sensitivity and specificity across AMD, DME, and normal categories [28]. These advancements underscore the growing role of SL in enhancing OCT analysis, contributing to accurate and efficient diagnostics in retinal healthcare.

A major challenge in deploying SL models for disease detection lies in the limited availability of labeled data, especially for rare or specific conditions. SL algorithms require large labeled datasets to achieve robust performance. The process of annotating and diagnosing data is resource-intensive and time-consuming for medical professionals. While routine screenings can generate raw data, labeling these datasets remains a barrier. Semi-supervised learning (SSL) approaches offer a promising solution by leveraging large amounts of unlabeled data alongside smaller labeled datasets to build effective classifiers. This makes developing automated systems capable of assessing and distinguishing diseases with minimal labeled data essential for advancing medical diagnostics.

Recent studies highlight the challenge of limited labeled data in medical imaging, particularly for rare or early-stage eye diseases, and emphasize the potential of SSL in addressing this issue. SSL has been widely explored for detecting retinopathies in OCT and fundus images [29,30]. Dali Chen et al. proposed an SSL approach combining U-Net with an iterative dataset updating strategy, improving blood vessel segmentation in retinal images [31]. Amir Rahdar et al. leveraged unlabeled data for retinoblastoma tumor segmentation, using a Gaussian mixture model (GMM) to detect abnormalities in fundus images [32]. Additionally, Andres Diaz-Pinto et al. improved glaucoma prediction accuracy by integrating a Deep Convolutional Generative Adversarial Network (DCGAN) with an SSL-based retinal image synthesizer, achieving an AUC of 0.9017 on 86,926 retinal images [33]. Xi Wang et al. employed a Student-Teacher architecture for diabetic macular edema (DME) detection in OCT images, utilizing confidence-based pseudo-labeling and consistency regularization to address label noise [34]. Further, Sixu Duan et al. proposed a semi-supervised graph-attentional convolutional neural network (GACNN), combining CNN and graph convolutional networks (GCNs) with attention mechanisms to leverage both labeled and unlabeled fundus images [35]. Zhicong Tan et al. implemented a transfer learning method with sub-domain adaptation (TLSDA), achieving impressive classification accuracies of 93.63% and 96.59% on private datasets, outperforming conventional supervised approaches. These studies collectively demonstrate the efficacy of SSL in improving retinal disease classification using limited labeled data [36].

Despite the advancements in deep learning (DL) for AMD classification using OCT images, most existing models rely on large, well-annotated datasets, which are often challenging to obtain in medical imaging. The effectiveness of SSL in handling limited labeled data has been explored, but its optimal application for AMD classification remains underexplored. This study addresses this gap by systematically evaluating SSL performance under varying degrees of data scarcity, aiming to establish an effective strategy for leveraging unlabeled data in AMD diagnosis.

The primary aim of this study is to investigate methodologies for evaluating the diagnosis of diseases such as neurological disorders, Alzheimer’s disease, or eye diseases like Retinitis Pigmentosa (RP), for which very limited datasets are available, in order to propose an optimal performance of DL models, particularly SSL models. To achieve this, various experiments are conducted based on the use of a small dataset of OCT images to identify AMD diseases, including cases where the dataset is moderately reduced, slightly reduced, severely limited, and minimally available. This investigation is conduced across four categories, 70%, 50%, 20%, and 5% of the training dataset volume, to introduce the best SSL method based on the results. In this study, DL models are evaluated on 16,822 OCT scans of Noor Eye Hospital (NEH) to reach an optimized model for discriminating normal, dry and wet AMD.

## 2. Material and Methods

### 2.1. Database

This study utilized the publicly available Noor dataset, which consists of 16,822 retinal OCT images collected from 441 patients, comprising a total of 554 OCT volumes. The dataset covers various stages of age-related macular degeneration (AMD). Of the 16,822 B-scans, 8584 images are normal, 4998 depict drusen, and 3240 represent cases of choroidal neovascularization (CNV). At the volume level, there are 187 normal cases, 194 drusen cases, and 173 CNV cases out of the 554 total volumes. The OCT images were captured using the Heidelberg SD-OCT imaging system at Noor Eye Hospital and annotated by retinal specialists. The dataset includes patients aged 50 years or older with no other retinal pathologies and with good image quality (Q ≥ 20). Some patients had both eyes scanned, with each OCT volume containing an average of 30 B-scans.

### 2.2. Data Preprocessing and Augmentation

During the preprocessing stage, all images are resized to a consistent dimension to ensure uniform input for the model, facilitating efficient training. Additionally, normalization is applied to all images to standardize pixel intensity values, ensuring a zero mean and unit variance. This normalization step helps accelerate the convergence of the model during training by ensuring the inputs are on a comparable scale. To further enhance the diversity of the dataset and improve the model’s generalization ability, several data augmentation techniques are employed. These include random cropping to simulate different field-of-view variations, horizontal flipping to increase variability in orientation, and rotation within a controlled range. Additionally, affine transformations such as translation and shear are applied to simulate spatial variations, further enriching the training set. These augmentations help improve the model’s robustness by exposing it to a wider range of potential input variations, thereby reducing the risk of overfitting and enhancing its performance on unseen data. The augmentation techniques used in this study were selected based on findings from our previous research [22]. In that study, we systematically examined the effects of various augmentation methods on model performance and determined that these transformations effectively enhanced generalization without altering key anatomical features in OCT images.

### 2.3. Proposed Model

The aim of the first step of this study is to achieve best performance to distinguish mentioned dataset OCT images into normal, drusen, and CNV categories using SL models. Among the SL models, EfficientNet B0, part of the EfficientNet family of deep learning models, has been employed as a supervised learning model to classify different stages of AMD due to its design and architecture benefits. Our selection of EfficientNet B0 was motivated by the results of our prior work [22], where we compared different convolutional neural network architectures. EfficientNet B0 is specifically optimized to achieve a good balance between model accuracy and computational efficiency. This means it can deliver competitive performance on tasks while requiring less computational resources compared to larger models. The architecture of EfficientNet is designed to scale efficiently to different sizes (B0 to B7), allowing flexibility in choosing a model size based on the available computing resources and the specific requirements of the task. Also, due to pre-training on large datasets like ImageNet, it can be effectively used for transfer learning. This allows for faster training convergence and better performance on new tasks with smaller datasets. Moreover, it has demonstrated good generalization capabilities, meaning it can perform well across a variety of domains and datasets, making it versatile for different computer vision tasks [37,38]. Furthermore, based on several experiments and investigating the other models including ResNet, EfficientNet B0, and Attention, it has been concluded that the model exhibits superior performance in AMD classification on the NEH dataset [22].

In the next stage, only a defined portion of the training dataset volume is considered as labeled data with the remainder being unlabeled data. The SL model is trained on this labeled dataset and classified OCT images. The accuracy percentage of disease detection in each image by the model in this stage is crucial. For training the SSL model, the Teacher = Student method has been utilized. The Teacher-Student (TS) approach is a widely used strategy in SSL, aiming to enhance model performance by utilizing both labeled and unlabeled data. This method involves two distinct neural networks: the teacher and the student. Typically, the teacher network is a pre-trained model that generates pseudo-labels for the unlabeled data. The student network, which is undergoing training, learns from both the valid labeled data and the pseudo-labels produced by the teacher [39]. Initially, the teacher network is trained on the labeled dataset. Once trained, it predicts labels for the unlabeled data, and the confidence in these predictions helps create pseudo-labels. The confidence parameter essentially controls how much trust is placed in the pseudo-labels generated by the teacher model for the unlabeled data. Each prediction comes with an associated confidence score, which reflects how certain the teacher model is about the predicted label. The confidence parameter sets a threshold for these confidence scores. Only the pseudo-labels that exceed this threshold are used for training the student model. This helps ensure that only high-confidence (more reliable) pseudo-labels are incorporated into the student model’s training process. The primary purpose of the confidence parameter is to balance the trade-off between the quantity and quality of the pseudo-labels used for training. By carefully adjusting this parameter, the SSL process aims to improve the student model’s accomplishment by leveraging the additional data while minimizing the risk of learning from incorrect or uncertain labels.

The student network is subsequently trained using both the labeled and pseudo-labeled data, with the goal of improving classification performance. This technique capitalizes on the availability of unlabeled data, which are often easier and more cost-effective to obtain than labeled data. Utilizing pseudo-labels allows the student network to potentially generalize better to new, unseen data. The success of the student network is significantly dependent on the quality of the pseudo-labels provided by the teacher network, as low-quality pseudo-labels can hinder training. Therefore, it is essential to maintain a balance in the performance between the teacher and student networks to prevent issues such as overfitting.

**Figure 2:**
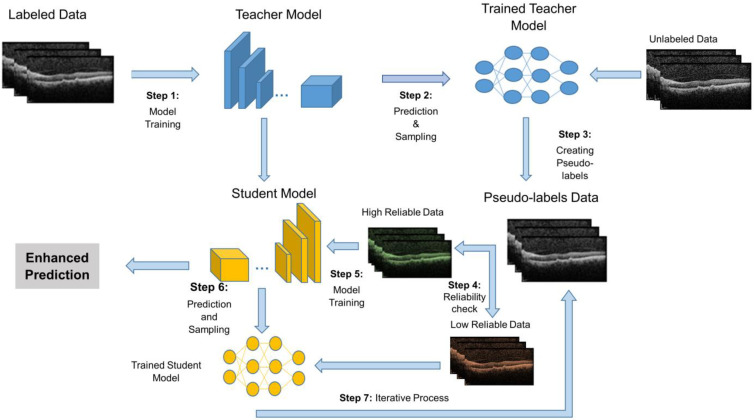
Overview of the proposed Iterative Teacher-Student (ITS) semi-supervised framework. The process begins with training the teacher model using labeled OCT images. Once trained, the teacher model predicts labels for unlabeled images, generating pseudo-labels based on high-confidence predictions. A reliability check is applied to filter out low-confidence pseudo-labels, ensuring that only reliable pseudo-labeled data are used for training. The student model is then trained on both labeled and high-confidence pseudo-labeled data. After training, the student model refines its predictions and is iteratively updated to improve performance. This iterative process enhances the classification accuracy by progressively leveraging unlabeled data while maintaining the reliability of pseudo-labels.

In this study, an Iterative Teacher-Student (ITS) approach is employed to enhance the training of deep learning models for OCT image analysis. The process begins with training a teacher model on the labeled portion of the dataset. The teacher model then generates pseudo-labels for the unlabeled OCT images, which are used to expand the training set. To ensure the reliability of the pseudo-labels, a confidence threshold is applied; only pseudo-labels with a confidence score above a certain threshold are added to the labeled dataset for the next iteration. This threshold helps ensure that only high-confidence predictions are incorporated into the training data, improving the quality of the pseudo-labels and preventing the model from learning from uncertain or incorrect labels. The student model is trained on both the original labeled data and the pseudo-labeled data from the teacher, refining its parameters through multiple iterations. This iterative process allows the model to progressively improve by leveraging unlabeled data, while the confidence threshold maintains control over the quality of the pseudo-labels used in each iteration, enhancing the model’s generalization capabilities and performance on the AMD classification task. Figure 2 provides a visual representation of the ITS entire process.

Below is a step-by-step explanation of Figure 2 to clarify the proposed semi-supervised framework (ITS):

- Step 1: Model Training (Teacher Model):
  ○ The process begins by training a Teacher Model using a labeled dataset (OCT images with known classifications: normal, drusen, and CNV);
  ○ The selected model (EfficientNet B0) is trained in a supervised manner to ensure accurate classification.

- Step 2: Prediction and Sampling (Teacher Model)
  ○ Once trained, the Teacher Model is used to predict labels for unlabeled data;
  ○ The model generates probabilistic predictions (classification scores) for these unlabeled OCT images.

- Step 3: Creating Pseudo-labels
  ○ The model assigns pseudo-labels to the unlabeled images based on its predictions;
  ○ These pseudo-labeled samples serve as additional training data for the next stage.

- Step 4: Reliability Check
  ○ A confidence threshold is applied to evaluate the reliability of the pseudo-labels;
  ○ Pseudo-labels with high confidence are accepted for training the Student Model;
  ○ Low-confidence pseudo-labels are set aside to avoid training errors.

- Step 5: Model Training (Student Model)
  ○ A Student Model is trained using a combination of the original labeled data and high-confidence pseudo-labeled data;
  ○ This enhances the model’s ability to generalize while utilizing additional unlabeled samples.

- Step 6: Prediction and Sampling
  ○ The trained Student Model is used to predict new labels;
  ○ These new predictions are incorporated into the training process, further refining the model.

- Step 7: Iterative Process
  ○ The Iterative Teacher-Student (ITS) approach is applied, where the refined Student Model can be used as a new Teacher Model for further iterations.

## 3. Experiments

To enhance computational efficiency, manage memory usage, and facilitate effective preprocessing and data augmentation in deep learning networks, all OCT images are resized to a uniform size of 224 × 224 pixels. Various batch sizes (16, 32 and 64) are tested to optimize computational resources and ensure the model generalizes well to new data. The training process typically spans 100 epochs to refine model parameters and avoid overfitting, with strategies such as early stopping, model checkpointing, and learning rate reduction (with a patience of 15 epochs) employed to preserve the best model. Data augmentation is applied to improve model performance, reduce overfitting, and enhance generalization. Additionally, the models are initialized with pre-trained weights from large image datasets, enabling faster convergence and transfer learning for AMD classification. The Adam optimizer, known for its adaptive learning rate capabilities, is used alongside categorical cross-entropy loss for multi-class classification tasks.

For model training, the dataset is divided into training, validation, and test sets, consisting of 10,811 training images, 2839 validation images, and 3235 test images from a total of 16,822 OCT images. The test set remains constant across all experiments to ensure consistent evaluation, while training data vary based on the strategies investigated and dataset sizes. To prevent information leakage, data are partitioned patient-wise, ensuring no overlap between the training and test sets. If class imbalance exists, appropriate techniques (e.g., oversampling or class weighting) are applied to address it. Data augmentation is employed on the training set to enhance model generalization.

In the second phase of the evaluation, the model’s performance is assessed using progressively smaller subsets of the dataset. Specifically, subsets containing 70%, 50%, and 20% of the original training data are created, with the remaining data excluded from the training process. The SL model, configured with parameters (obtained in the previous phase), is then trained and tested on these subsets. During this phase, various parameter configurations are explored to identify the optimal settings based on performance metrics. The impact of reducing the training dataset size on model performance is analyzed, and the results are compared to those from the later SSL phase to evaluate the added value of the semi-supervised approach.

In the SSL phase, a confidence threshold for labeling images is defined at 0%, 90%, 95% and 99% for each unlabeled image. The quantity and quality of the pseudo-labeled data significantly influence model performance. The confidence threshold levels were chosen to explore the trade-off between data utilization and label reliability in our SSL approach. A lower threshold (0%) allows the model to leverage a larger amount of unlabeled data, though it may introduce noisy pseudo-labels. In contrast, higher thresholds (90%, 95%, 99%) ensure that only high-confidence predictions contribute to training, reducing potential label noise. This stepwise approach enables us to assess how different confidence levels impact model performance and generalization. In contrast to the second phase, the model is presented with a mix of labeled and unlabeled data, where labeled images account for 70%, 50%, 20% and 5% of the total training data, while the rest are treated as unlabeled. As the model makes predictions on the unlabeled images, those with a confidence level exceeding the specified threshold are added to the labeled set as pseudo-labeled images. The model is then retrained with this expanded dataset to evaluate the effectiveness of the SSL approach in improving accuracy as more data become available.

Table 1 summarize the key hyperparameters and their values used in the experiments:

To further validate our model’s generalizability, we collected an independent dataset from the Eye Surgery Department of Mehr Hospital at Tabriz, Iran, consisting of 1000 OCT images per class (CNV, Drusen and Normal). These images were labeled by expert ophthalmologists and were not incorporated into the training process. Instead, they were used exclusively for testing to assess the model’s performance on real-world data. The same preprocessing steps applied to the original dataset were also used for this dataset to ensure consistency in evaluation.

## 4. Results

### 4.1. SL Training Phase

Initially, the complete dataset was used to train the SL model, optimizing key parameters such as batch size, data augmentation, and the inclusion of pre-trained weights. The experiments showed no significant performance differences across batch sizes, although smaller sizes increased training time. Based on these results, a batch size of 64 was selected as it provided a good balance between performance and training efficiency. Transfer learning substantially improved classification accuracy compared to models trained from scratch, highlighting its role in leveraging pre-trained feature representations. Moreover, fine-tuning pre-trained layers yielded a 7–10% increase in detection accuracy over freezing them, underscoring the importance of adaptable feature extraction for disease classification. Table 2 summarizes the SL model’s performance under different parameter settings.

### 4.2. Limited Data in SL Models

**Figure 3:**
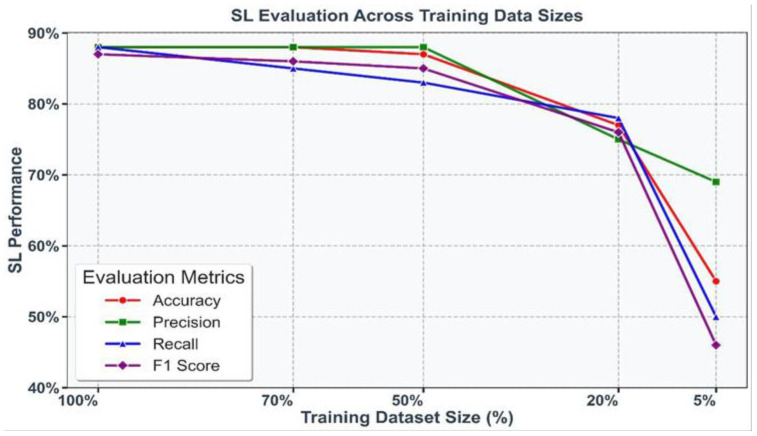
Performance of SL models (accuracy, precision, recall, and F1 score) under varying training dataset sizes (100%, 70%, 50%, 20% and 5%). Each metric is represented by a unique color, illustrating the decline in performance with reduced data availability.

To evaluate the impact of data scarcity, SL models were trained on progressively smaller subsets of the training dataset: 100%, 70%, 50%, 20% and 5%. A consistent decline in performance was observed as the data volume decreased, with accuracy dropping sharply from 88.44% at 70% of the dataset to 77.05% at 20%, and further reductions seen with only 5% of the data. Similar trends were evident across precision, recall, and F1 score, underscoring the sensitivity of SL models to training data size. These results are visualized in Figure 3, where each metric is represented by a distinct color, highlighting the critical importance of data availability in maintaining robust model performance.

### 4.3. Performance of Teacher-Student (TS) Models

The TS approach was evaluated using datasets with varying proportions of labeled data (70%, 50%, 20% and 5%) alongside unlabeled data. Pseudo-labeling was employed to generate additional labeled examples, with different confidence thresholds determining the inclusion criteria for pseudo-labeled images.

Based on the results from all the experiments, it is observed that the best outcomes were achieved with a 95% confidence criterion when the dataset size was reduced to 20%. The accuracy at 70% was 88.71, and at 50% it was 88.32. Furthermore, defining a confidence threshold of 95% significantly enhances the model’s achievement across all metrics: accuracy, precision, recall, and F1 score. This improvement indicates that the integration of a substantial volume of pseudo-labeled data, even with lower confidence, positively impacts model capabilities. Performance at the 20% dataset size across various metrics is shown in Table 3.

With a significant reduction in dataset size to 20% and 5%, the model’s diagnostic competency obviously diminished. However, by increasing the strictness of the confidence criterion, particularly at the 5% dataset size, better results were achieved. At this reduced size, with a confidence level of 95%, the accuracy was 49.34%, and with a 99% confidence level, the accuracy improved to 55.88%.

### 4.4. Performance of Iterative Teacher-Student (ITS) Models

**Figure 4:**
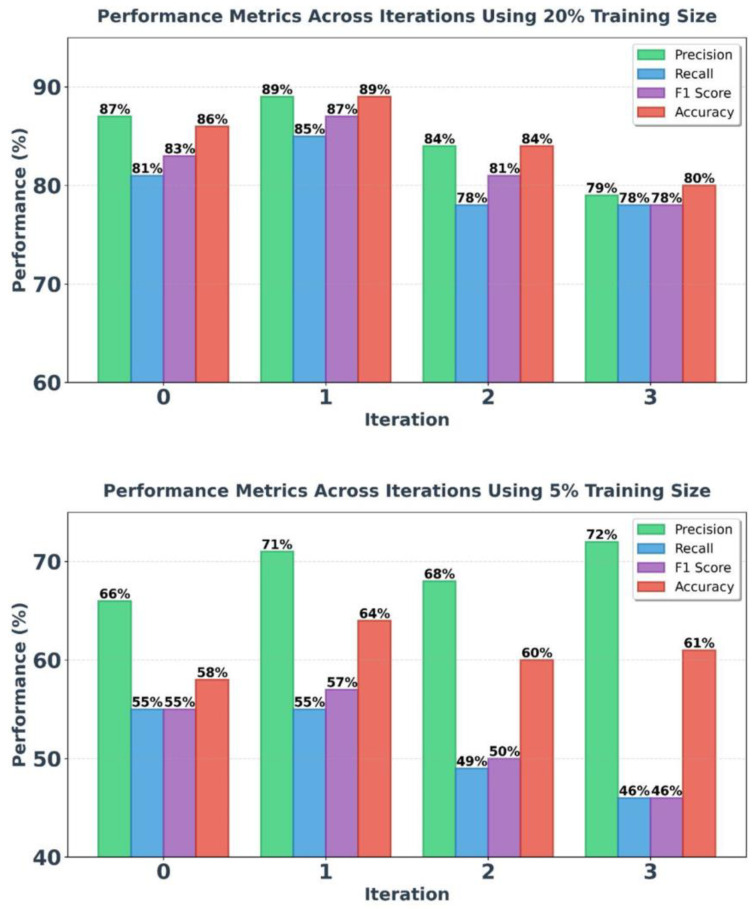
ITS model results at 20% and 5% of dataset volume in four iterations.

The proposed ITS model, considering the improvement of the TS model’s results by adding pseudo-labeled data, aims to refine and enhance the model’s detection quality in performance metrics. Applying this method can improve the severe decrease in accuracy at lower volumes, especially in specific diseases with limited labeled data. Based on the results obtained from the TS model, the best model performances at 20% and 5% volumes have been included in the iteration process. Refer to Figure 4 for the results of the iteration process at 20% and 5% of the training dataset.

Based on the experimental results, the model’s efficiency in the iterative process positively affected its accuracy and precision. Compared to the TS model results, the diagnostic accuracy of the model at a 20% dataset volume shows a 2.5% increase in the first iteration. Additionally, at a 5% dataset size, an upward trend is observed in the iterative process, particularly in the second iteration, where the model’s accuracy increased by 10%, reaching 64.15%.

The iterative process demonstrates that while the addition of high-confidence pseudo-labels initially improves model accuracy and reduces test loss, excessive pseudo-labeling can lead to diminishing returns and potential overfitting. The model performs best with a balanced amount of pseudo-labeled data, as seen in the first and second iteration, and shows decreased performance with further iterations.

### 4.5. Confusion Matrix

**Figure 5:**
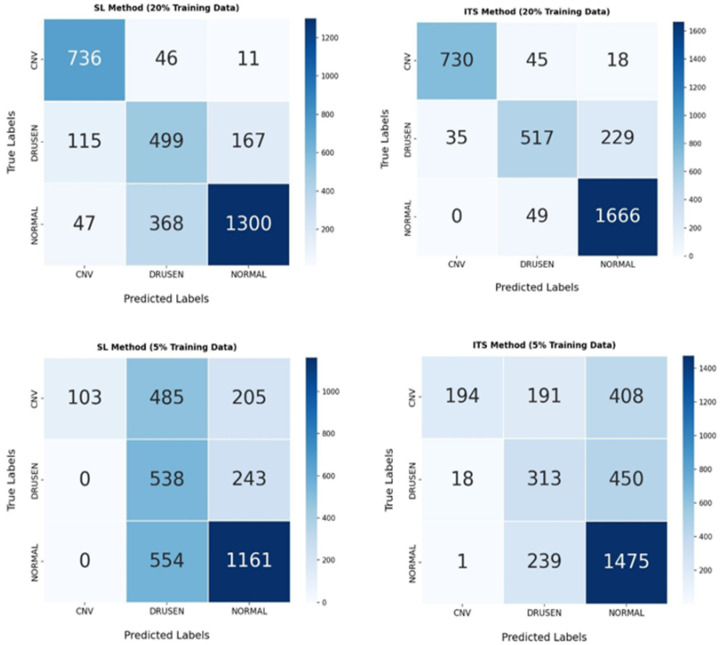
Confusion matrices for model evaluation: true vs. predicted labels across three AMD classes at 20% and 5% of dataset size in SL and ITS models.

To provide a detailed assessment of the classification performance of each model, the four confusion matrices (CMs) corresponding to SL and our proposed ITS Model are presented. Each confusion matrix is a 3 × 3 table that displays the distribution of true labels versus predicted labels for the three AMD classes, normal, drusen, and CNV, across all datasets (see Figure 5).

The confusion matrices in Figure 5 highlight the challenges of AMD classification using a limited dataset. A high number of false positives (FPs) and false negatives (FNs) is expected in such scenarios due to the limited availability of labeled training data, making it difficult for the model to generalize effectively. Additionally, the inherent similarity between OCT images of different AMD stages and healthy individuals contributes to classification challenges, particularly in early-stage disease cases where subtle pathological changes may not be easily distinguishable. Notably, drusen cases were more frequently misclassified, likely because the visual features of drusen are often subtle and can overlap with normal variations, making them especially challenging to differentiate from both healthy and other early-stage AMD presentations.

However, our results demonstrate a clear improvement when using the ITS technique compared to the standard SL method. Specifically, ITS leads to a noticeable reduction in false predictions while increasing the number of correct classifications across all classes. This improvement suggests that leveraging unlabeled data via SSL helps the model better differentiate between classes, even with a small labeled dataset. Therefore, despite the challenges associated with OCT-based classification, our study highlights the potential of ITS to enhance performance in real-world clinical settings where labeled data are scarce.

### 4.6. ROC Curve Analysis

**Figure 6:**
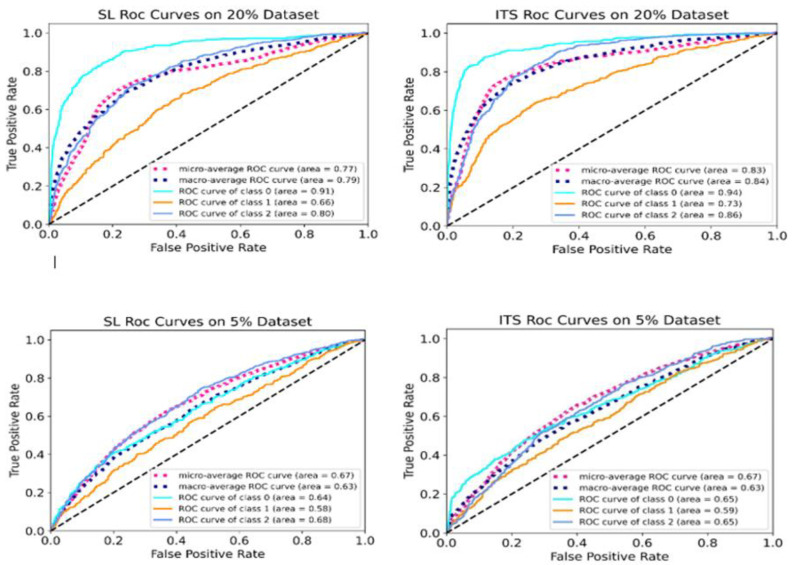
ROC curves illustrating SL and ITS model performance at 20% and 5% of dataset volume: individual class ROC curves (classes 0, 1, 2), micro-average ROC curve, and macro-average ROC curve. The dashed diagonal line represents a reference line (line of no-discrimination).

To thoroughly assess the performance of the optimized supervised learning (SL) model and the proposed iterative teacher-student (ITS) model in detecting and distinguishing AMD cases at reduced dataset volumes (20% and 5%), Figure 6 presents the receiver operating characteristic (ROC) curves. These include ROC curves for the AMD classes (normal, drusen, and CNV), along with the micro-average and macro-average ROC curves.

The micro-average curve combines the true positive and false positive rates across all classes, and then calculates performance metrics based on these combined totals. This provides a comprehensive assessment of the model’s overall performance and gives an insight into how both the SL and ITS models perform across all classes. In contrast, the macro-average curve computes the performance metrics like ROC curve for each AMD class individually, and then averages these values, giving equal weight to each class. By comparing these curves across both SL and ITS settings, we can evaluate the effectiveness of the proposed method in differentiating between AMD cases.

### 4.7. Generalizability of the Proposed Model

To evaluate the generalizability of our model beyond the labeled dataset, we tested its performance on the independent Mehr Hospital dataset. When trained on the entire dataset, the model achieved an accuracy of 86.7% on this external dataset. However, when trained on only 20% of the labeled dataset, performance dropped to 79.9%, highlighting the impact of limited training data. After applying the ITS method, accuracy improved to 83.2%, demonstrating its effectiveness in reducing false predictions and enhancing classification reliability. These results suggest that our approach maintains robustness even when tested on real-world clinical data that was not seen during training.

## 5. Discussion

The study demonstrates the critical role of parameter selection, transfer learning, and data augmentation in achieving optimal performance in supervised learning (SL) models for OCT image classification. Experiments revealed that batch size had no significant impact on performance but influenced training time, with smaller batch sizes increasing computational costs. Incorporating transfer learning significantly enhanced classification accuracy, particularly when pre-trained layers were fine-tuned. Models trained with frozen pre-trained layers exhibited a 7–10% decrease in accuracy, emphasizing the importance of enabling further learning within these layers. The SL model achieved its best performance with a batch size of 64, transfer learning, unfrozen pre-trainable layers, and effective data augmentation.

As the training dataset size decreased from 70% to 20% of the total data, a consistent decline in performance metrics, including accuracy, precision, recall, and F1 score, was observed. Accuracy dropped from 88.44% with 70% of the training data to 77.05% with 20%, highlighting the sensitivity of model performance to dataset size. Despite this, the robustness of the chosen parameter settings across varying data volumes was evident. However, the results underscore the need for larger training datasets to ensure optimal model performance, particularly in scenarios where high accuracy is critical. Decision-makers must carefully weigh the trade-offs between computational efficiency and model performance when determining dataset sizes.

The findings also underscore the importance of pre-trainable layers in SSL models. The inclusion of these layers led to significant improvements across all metrics, as they enhanced the model’s ability to generalize and make accurate predictions. For SSL models, unfrozen pre-trainable layers contributed to a 14.05% improvement in accuracy compared to frozen layers, a greater benefit than the 5.40% gain observed in SL models. This suggests that SSL methods are better equipped to leverage additional fine-tuning, making them more adaptable to exploiting learnable parameters and improving overall performance.

In the TS model, when the dataset is reduced, the 95% confidence level can be effective. However, when the dataset size is drastically reduced, increasing the strictness of the confidence threshold yields better results.

Pseudo-labeling in SSL was shown to be an effective strategy for leveraging unlabeled data, with accuracy improving from 77% in the teacher model to 82.15% in the student model using pseudo-labeled data. The iterative process revealed that a balanced approach to adding high-confidence pseudo-labels was essential for maintaining model performance while minimizing test loss. Excessive pseudo-labeling led to diminishing returns and potential overfitting, emphasizing the need for careful management of pseudo-labeling strategies. Despite these challenges, the results demonstrated that pseudo-labeling, even with lower-confidence data, positively impacted model performance by providing valuable supplementary information.

Overall, this study highlights the distinct benefits of SSL over SL, particularly in scenarios where labeled data are limited. While both SL and SSL benefit from unfrozen pre-trainable layers, SSL shows a more pronounced improvement, suggesting its superior adaptability to fine-tuning. The findings emphasize the importance of careful parameter tuning, dataset size considerations, and pseudo-labeling strategies in optimizing model performance. These insights can guide future research and practical applications, paving the way for more effective machine learning systems in disease classification and beyond.

The proposed ITS framework has significant potential for real-world applications in clinical settings. For instance, it could be integrated into ophthalmology clinics as a decision-support tool, assisting clinicians in diagnosing retinal diseases such as AMD, particularly in resource-constrained environments where labeled data are scarce. The framework could also be deployed in telemedicine platforms, enabling remote diagnosis and monitoring of patients in rural or underserved areas. By automating the analysis of OCT images, the framework could reduce the time required for manual interpretation, allowing clinicians to focus on treatment planning and patient care. Furthermore, the ITS framework could be adapted for use in screening programs, enabling early detection of retinal diseases and improving patient outcomes.

While the proposed framework shows promising results, several limitations must be acknowledged. The Noor dataset, although comprehensive, may have inherent biases due to its demographic composition (e.g., age, ethnicity, and geographic location), which could limit the generalizability of the results to other populations. Additionally, variations in imaging protocols and device specifications across different healthcare facilities may affect the performance of the framework. To address these challenges, future studies should validate the framework on multi-center datasets with diverse patient populations and imaging protocols. This would help ensure the robustness and generalizability of the proposed approach across different clinical settings.

To further advance the field, several future research directions are proposed. First, investigating additional deep learning architectures, such as Vision Transformers (ViTs) or hybrid models combining CNNs with attention mechanisms, could improve the accuracy of retinal disease diagnosis. Second, exploring methods to enhance the quality of pseudo-labels in smaller datasets, such as incorporating uncertainty estimation techniques or leveraging multi-modal data (e.g., combining OCT images with clinical metadata), could further refine the ITS framework. Third, reducing the computational cost and memory requirements of the framework would make it more feasible for deployment in real-world clinical settings. Finally, conducting longitudinal studies to evaluate the long-term performance of the framework in clinical practice, including its impact on patient outcomes and healthcare costs, would provide valuable insights into its effectiveness and scalability.

By addressing these challenges and exploring these future directions, the proposed ITS framework has the potential to significantly improve the diagnosis and management of retinal diseases, ultimately enhancing patient care and outcomes.

## Figures and Tables

**Table 1:** Summary of key hyperparameters and their values used in the experiments.

Hyperparameter	Value/Range	Description
Image Size	224 × 224 pixels	All OCT images are resized to this uniform size for consistency.
Batch Size	16, 32, 64	Tested to optimize computational efficiency and generalization.
Epochs	100	Total number of training iterations.
Early Stopping Patience	15 epochs	Training stops if no improvement is observed for 15 epochs.
Learning Rate	0.001	Initial learning rate for the Adam optimizer.
Learning Rate Reduction	Factor: 0.1, Patience: 10 epochs	Learning rate is reduced by a factor of 0.1 if no improvement is seen.
Optimizer	Adam	Adaptive learning rate optimizer used for training.
Loss Function	Categorical Cross-Entropy	Used for multi-class classification of AMD stages.
Data Augmentation	Random cropping, flipping, rotation	Techniques applied to enhance dataset diversity and reduce overfitting.
Transfer Learning	Pre-trained EfficientNet-B0 (ImageNet)	Model initialized with pre-trained weights for faster convergence.
Confidence Thresholds	0%, 90%, 95%, 99%	Thresholds for pseudo-labeling in the SSL phase.
Training Data Split	70%, 50%, 20%, 5%	Subsets of the training data used to evaluate performance under data scarcity.
Validation Split	20% of training data	Used for monitoring model performance during training.
Test Set	3235 images	Constant across all experiments for consistent evaluation.
Class Weighting	Adjusted for class imbalance	Applied to address class imbalance in the dataset.

**Table 2:** Performance metrics of SL model using optimizing parameters. The best results are highlighted in bold.

Experiment Number	Transfer Learning	Pre-Trained Layers	Data Aug.	Accuracy (%)	Precision (%)	Recall (%)	F1 Score (%)
**1**	**ImageNet**	**Fine-tuned**	**Enabled**	**87.14**	**87.33**	**87.66**	**87.33**
2	ImageNet	Frozen	Enabled	77.31	76.66	75.33	76.66
3	ImageNet	Fine-tuned	Disabled	86.27	88.33	84.66	86
4	Random	Fine-tuned	Enabled	86.27	86.66	85.33	85.66

**Table 3:** Performance metrics changes in 20% of training dataset. The best results for each metric are highlighted in bold.

Model	Acc. (%)				Prec. (%)				Recall (%)				F1-Sco. (%)			
	Confidence (%)				Confidence (%)				Confidence (%)				Confidence (%)			
	0	90	95	99	0	90	95	99	0	90	95	99	0	90	95	99
Trained on 20%	82.15	84.25	**86.01**	85.31	84	85	**87.33**	81.66	77	81	**86.01**	80	79	**82**	81	80

## Data Availability

The data presented in this study are openly available at: https://data.mendeley.com/datasets/8kt969dhx6/1 accessed on 6 October 2021.
